# The Carcinogenic Activity of 2-Naphthylamine

**DOI:** 10.1038/bjc.1956.62

**Published:** 1956-09

**Authors:** Georgiana M. Bonser, D. B. Clayson, J. W. Jull, L. N. Pyrah

## Abstract

**Images:**


### THE CARCINOGENIC ACTIVITY OF 2-NAPHTHYLAMINE

GEORGIANA M. BONSER, D. B. CLAYSON, J. W. JULL
AND L. N. PYRAH

*From the Departments of Experimental Pathology and Cancer Research, and of Urology, School of Medicine, Leeds*

Received for publication June 21, 1956

HUEPER (1938) and Bonser (1943) established that carcinoma of the bladder may be induced by feeding 2-naphthylamine to dogs over a protracted period. In the more recent experiments of Bonser, Clayson, Jull and Pyrah (1952) the oral administration of 2-naphthylamine to mice resulted in a significant yield of liver tumours, but in 5 rabbits which survived 4 years of treatment the only changes induced were a small papilloma of the bladder in one and advanced epithelial hyperplasia in another. Bonser, Clayson and Jull (1951) presented evidence that 2-naphthylamine is carcinogenic by virtue of its conversion in the body to 2-amino-1-naphthol. On the basis of these findings, a hypothesis covering the mode of action of the aromatic amines in general has been put forward (Clayson, 1953). However, doubts as to the validity of these postulates have persisted due in part to the fact that in a few cases the injection of oily solutions of 2-naphthylamine (Hackmann, 1951) or of extracts of the chemical (Case and Pearson, 1952) has resulted in the appearance of subcutaneous sarcomas. Thus there was the possibility that the carcinogenic activity of 2-naphthylamine was due to an impurity present to a small extent in the substance used by Bonser (1943) and to a greater extent in the crude industrial material used by Hueper (1938).

The following experiments were carried out to test the local and distant carcinogenic properties of 2-naphthylamine following its subcutaneous injection into mice, and to discover whether the chemical, highly purified by gradient sublimation, as developed by Dr. R. A. M. Case (*cf* Henson, Somerville, Farquharson and Goldblatt, 1954), was carcinogenic to dogs.

### MATERIALS

2-*Naphthylamine* purified as described by Bonser (1943) and supplied by British Drug Houses Ltd., was termed BDH.

2-*Naphthylamine* purified by the process of gradient sublimation described by Henson *et al.* (1954) was stored before use at 0° C. and was termed RCH.

Mongrel dogs were used.

All the organs were examined *post mortem* and the bladder, liver and kidneys were examined microscopically. The bladder of one dog (86) was examined by cystoscope during life.

For experiments 2–4, Swiss type albino mice, approximately 12 weeks of age, were obtained in one batch from a dealer. Some spontaneous tumours of lymphoid tissue and one ovarian and one breast tumour occurred in a control group injected with arachis oil only but no hepatomas were observed among 11 mice surviving for 70 weeks or more.

The CBA mice were from a colony inbred for over 70 generations. Bonser *et al.* (1952) reported an incidence of 8·1 per cent of hepatomas in breeding mice of this strain. During the period of the present investigation one hepatoma was observed in a female at 123 weeks of age among 5 male and 10 female breeding mice which survived more than 78 weeks. The mice were approximately 12 weeks of age at the commencement of treatment.

The livers of all the mice, both control and experimental, were examined microscopically. In all but a very few mice living for 50 weeks or more after the beginning of treatment there were small collections of inflammatory cells (polymorphs, lymphocytes and fibroblasts) in the portal tracts. In the livers of experimental mice in which hepatomas occurred, there was no greater degree of cellular infiltration of the portal tracts. There was, however, mild proliferation of the bile ducts in many of the livers and in a few there were small benign cholangiomatous areas.

*Experiment* 1

2-Naphthylamine (RCH) was given orally in a gelatine capsule 6 days a week to 4 female dogs. Initially the dose was 200 mg. but this was increased after 6 months to 600 mg. The maximum cumulative dose of chemical administered to a dog was 310 g.

*Dog* 87 (female) died after 14½ months of treatment with no neoplastic changes in the bladder.

*Dog* 95 (female) received 2-naphthylamine for 1 year only and was killed 1¾ years after the beginning of treatment. There were no neoplastic changes in the bladder epithelium.

*Dog* 89 (female) died after 2 years of treatment. There were over 40 transitional cell papillomas up to 2 cm. in diameter in the bladder (Fig. 1). Although some were regarded as histologically malignant none was invasive.

*Dog* 86 (female). On cystoscopic examination of the bladder 2 years after the beginning of treatment epithelial elevations and congestion were seen. Feeding of the chemical was then stopped. On re-examination ten months later two papillary tumours were seen, one of which was clinically malignant. This dog was killed 3 years after treatment had been started. At *post mortem* examination, many papillomas were present in the bladder, the picture being comparable with that in Dog 89. The tumours were of transitional-cell type. One was a frank carcinoma and was invading the muscle of the bladder wall.

*Experiment* 2

A 3 per cent solution of 2-naphthylamine (BDH) in arachis oil was allowed to stand for at least 4 weeks. 0·1 ml. of this solution was injected subcutaneously into albino mice twice weekly for 50 weeks.

Ten of 16 mice surviving 20 or more weeks of treatment developed subcutaneous sarcomas near the injection sites (Table I). Four of 5 mice dying after 80 weeks had hepatomas and in three there were areas of cholangioma (Table II).

*Experiment* 3

0·1 ml. of a 3 per cent solution of 2-naphthylamine (BDH) in arachis oil, prepared immediately before use, was injected subcutaneously into albino mice twice weekly for 50 weeks. None of 13 mice which survived 33 weeks or more of treatment developed sarcomas, but 2 of 4 mice living over 77 weeks had hepatomas (Tables I and II).

*Experiment* 4

The treatment was similar to that in Experiment 3, except that a freshly prepared solution of 2-naphthylamine (RCH) was used.

Local sarcomas occurred at 37 and 41 weeks of treatment in 2 of 12 mice surviving for 37–69 weeks. One cholangioma associated with cirrhosis was observed at 43 weeks and one small hepatoma at 52 weeks. The last mouse in this group died at 69 weeks, 8 weeks before the first hepatoma was seen in Experiments 2 or 3.

*Experiment* 5

0·1 ml. of a 3 per cent solution of 2-naphthylamine hydrochloride (RCH), freshly prepared in warm water, was injected subcutaneously into CBA mice twice a week for 6 months and then once a week for a further 4 months.

No sarcomas were observed in any of 10 mice surviving more than 58 weeks. Hepatomas were seen at 77 and 93 weeks in 2 of 8 mice dying after 70 weeks (Table II).

*Experiment* 6

The treatment was the same as in Experiment 5, except that 2-naphthylamine hydrochloride (BDH) was used.

No sarcomas occurred in any of the 11 mice surviving more than 56 weeks of treatment. Hepatomas were seen in 4 of 11 mice dying after 82 weeks.

### DISCUSSION

The induction of bladder tumours in the two dogs of Experiment 1 which lived for two years or more is evidence that 2-naphthylamine purified by the most stringent means is as potent a bladder carcinogen as the chemical used by Bonser (1943). A summary of the results of treatment with 2-naphthylamine in all the dogs investigated in Leeds since 1938 is given in Table III. Every dog treated for 2 years or longer has developed bladder tumours. Few other tests of carcinogens have yielded tumours in all the experimental animals. These facts would support the view that the carcinogen is concentrated in the urine. As yet there has been no report that tumours occur in other sites in dogs fed with 2-naphthylamine.

The experiments with mice, designed to test whether 2-naphthylamine is a local or distant carcinogen, or both, have shown that the incidence of local sarcomas can be greatly reduced by using freshly prepared solutions in oil, or by avoiding oily solutions altogether and using aqueous solutions of the hydrochloride. In

---

EXPLANATION OF PLATE.

FIG. 1.—Dog 89, female, oral 2-naphthylamine RCH for 2 years. Bisected bladder showing multiple papillomata, ranging in size from pinhead to 2 cm. diameter. Most of the tumours are slightly penduncúlated, but the largest one (not seen here) was sessile, with a hard base. × 1·5.

Bonser, Clayson, Jull and Pyrah.

TABLE III.—*Dogs treated with 2-naphthylamine by mouth*

| Dog No. | Sex. | Survival (years). | Source of chemical. | Bladder tumours. | Other observations. |
|---|---|---|---|---|---|
| I | M | 1 | BDH | –* | |
| 87 | F | $1\frac{1}{4}$ | RCH | – | |
| 2 | F | $1\frac{1}{2}$ | BDH | – | |
| 95 | F | $1\frac{3}{4}$ | RCH | – | Received chemical for one year only |
| 89 | F | 2 | ,, | + | |
| 86 | F | 3 | ,, | + | Received chemical for 2 years only |
| II | F | $3\frac{2}{3}$ | BDH | +* | |
| III | M | 4 | ,, | +* | |
| 1 | F | $4\frac{1}{4}$ | ,, | + | |
| 3 | M | $4\frac{1}{2}$ | ,, | + | |
| IV | M | 5 | ,, | +* | |
| 5 | F | 5 | ,, | + | Sodium bicarbonate daily |
| 6 | F | $5\frac{1}{4}$ | ,, | + | ,, ,, ,, |
| 7 | M | $5\frac{1}{4}$ | ,, | + | ,, ,, ,, |
| 4 | F | $5\frac{3}{4}$ | ,, | + | |

* Described by Bonser, 1943.

Experiment 2, in which 2-naphthylamine (BDH) in oily solution was injected after standing for several weeks, an incidence of 63 per cent of local sarcomas was obtained (Table I), but in Experiments 3 and 4 in which freshly prepared oily solutions of 2-naphthylamine (BDH and RCH) were used, the incidence of these tumours was reduced to 8 per cent. In Experiments 5 and 6, in which the aqueous hydrochloride was used, local sarcomas were eliminated altogether. It is deduced from these results that on standing oily solutions of 2-naphthylamine develop local carcinogenic properties. A similar change may well take place when freshly prepared oily solutions are introduced into the subcutaneous tissues, thus accounting for the small yield of sarcomas in mice which received such solutions.

In assessing the significance of the occurrence of hepatomas, it should be noted that these arose late, i.e. with one exception after the 70th week. From Experiments 5 and 6 it is apparent that hepatomas can be induced by 2-naphthylamine hydrochloride without accompanying sarcomas. The induction time of liver tumours in all experiments was, with one exception, between 77 and 91 weeks, but only one sarcoma occurred after 54 weeks. It is thus possible that the causative agent of the sarcomas in these experiments is different from that which induced the hepatomas. The hepatoma incidence in mice treated with 2-naphthylamine was 66 per cent after 70 weeks or more, and with 2-naphthylamine hydrochloride 33 per cent. These results are comparable with those reported by Bonser *et al.* (1952), within the limits of the small number of effective mice.

### SUMMARY

1. Oral administration of very pure 2-naphthylamine induced multiple tumours of the bladder in 2 dogs surviving 2 years or more of treatment.

2. Local sarcomas were induced in 63 per cent of mice injected subcutaneously with an oily solution of 2-naphthylamine which had stood for 4 weeks.

3. When freshly prepared oily solutions of 2-naphthylamine were similarly injected the incidence of local sarcomas was reduced to 8 per cent.

4. Subcutaneous injection of 2-naphthylamine hydrochloride did not induce local sarcomas.

5. Although spontaneous hepatomas occurred in control CBA breeding mice a greater incidence occurred in mice which received either specially purified or "partially" purified 2-naphthylamine in the form of injections in oil or as aqueous solutions of the hydrochloride.

6. It is concluded that 2-naphthylamine purified by gradient sublimation is carcinogenic to the dog and the mouse.

The 2-naphthylamine, purified by gradient sublimation, was a gift from the Chester Beatty (K. lab) through the kind offices of Professor F. Bergel, to whom the authors wish to express their thanks.

### REFERENCES

BONSER, GEORGIANA M.—(1943) *J. Path. Bact.*, **55**, 1.
*Idem*, CLAYSON, D. B. AND JULL, J. W.—(1951) *Lancet*, ii, 286.
*Iidem* AND PYRAH, L. N.—(1952) *Brit. J. Cancer*, **6**, 412.
CASE, R. A. M. AND PEARSON, J. T.—(1952) *Proc. IIe Congr. int. Biochem.*, p. 464.
CLAYSON, D. B.—(1953) *Brit. J. Cancer*, **7**, 460.
HACKMANN, C.—(1951) *Z. Krebsforsch.*, **58**, 56.
HENSON, A. F., SOMERVILLE, A. R., FARQUHARSON, MURIEL E. AND GOLDBLATT, M. W. —(1954) *Biochem. J.*, **58**, 383.
HUEPER, W. C. (1938) *Arch. Path.*, **25**, 856.


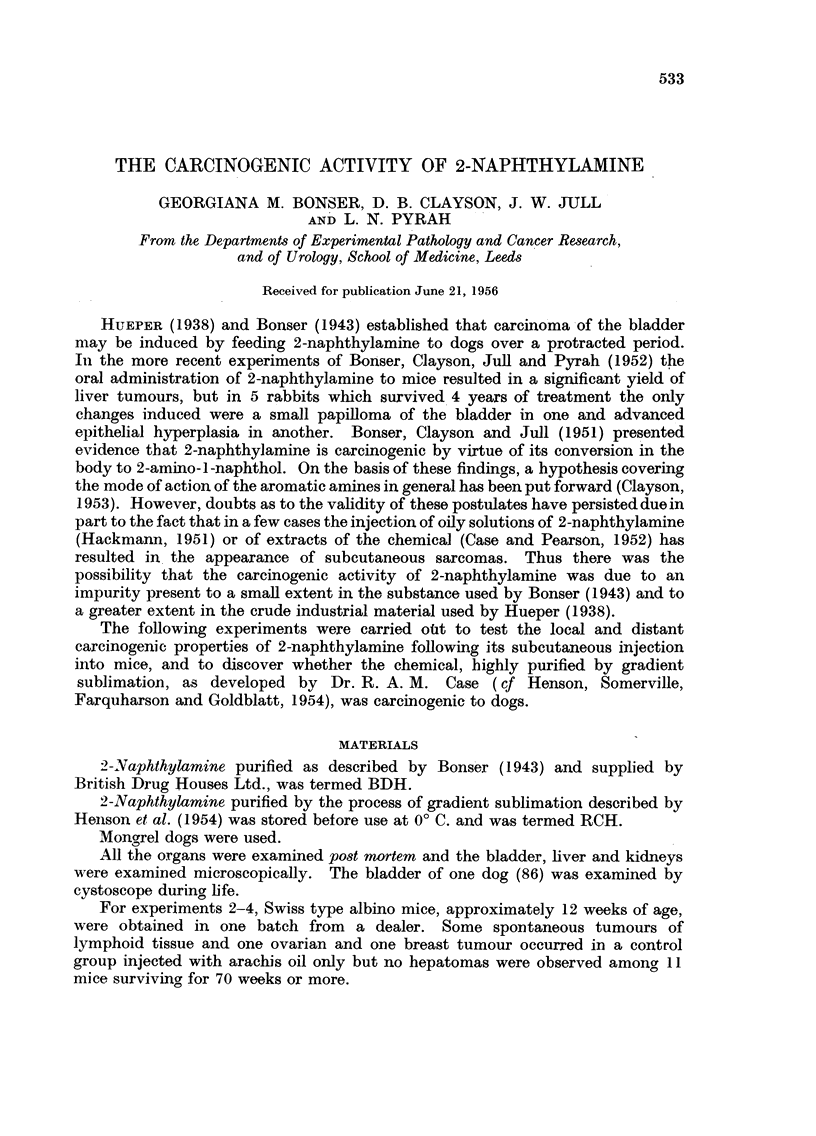

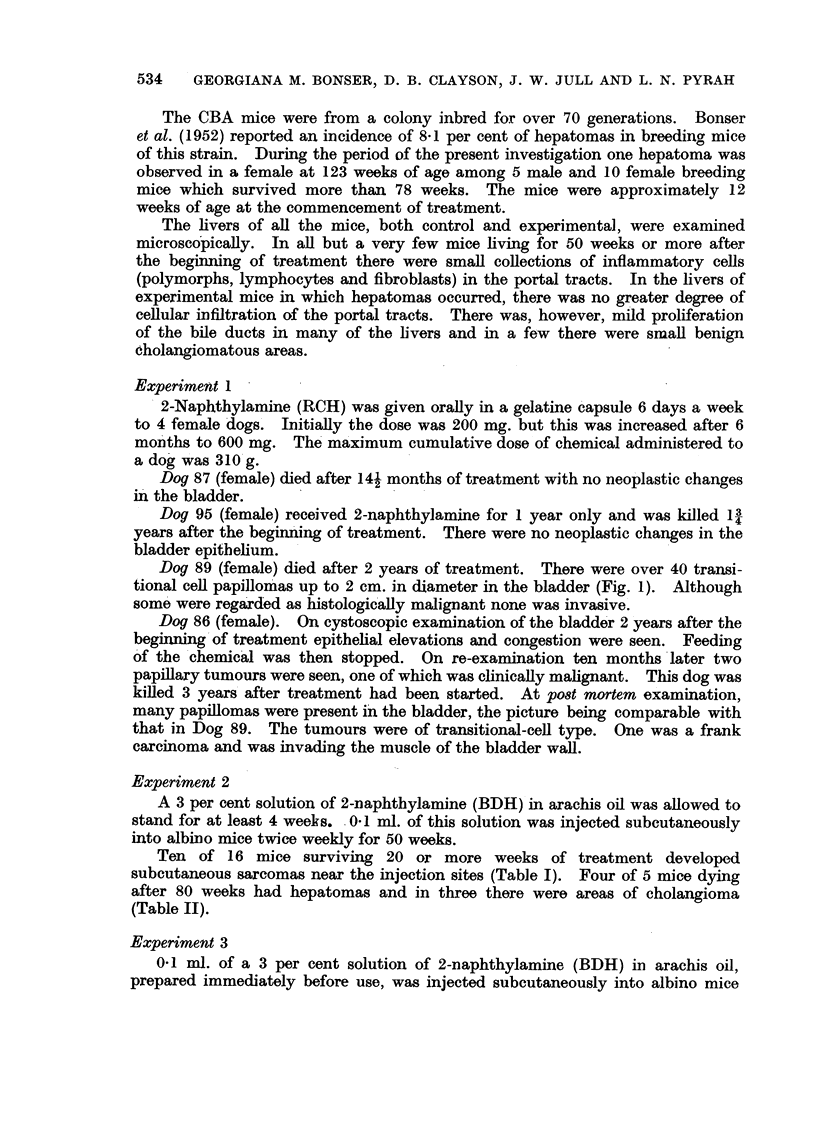

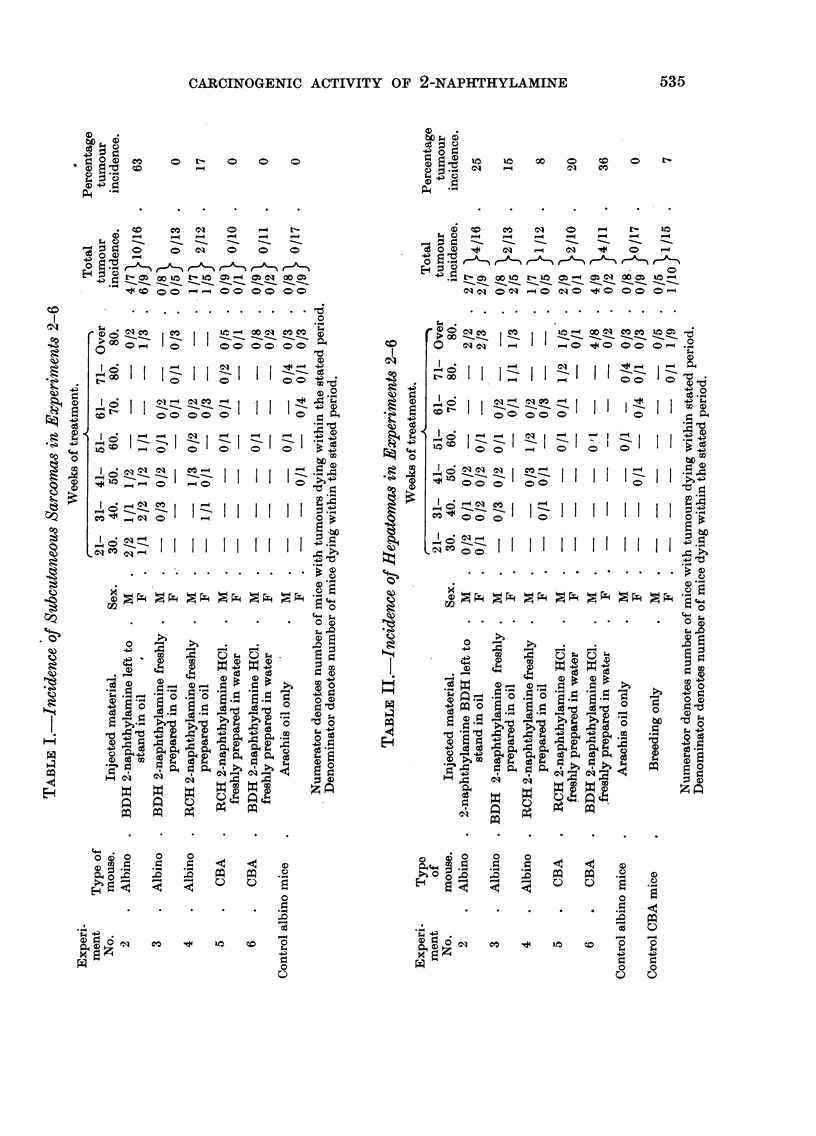

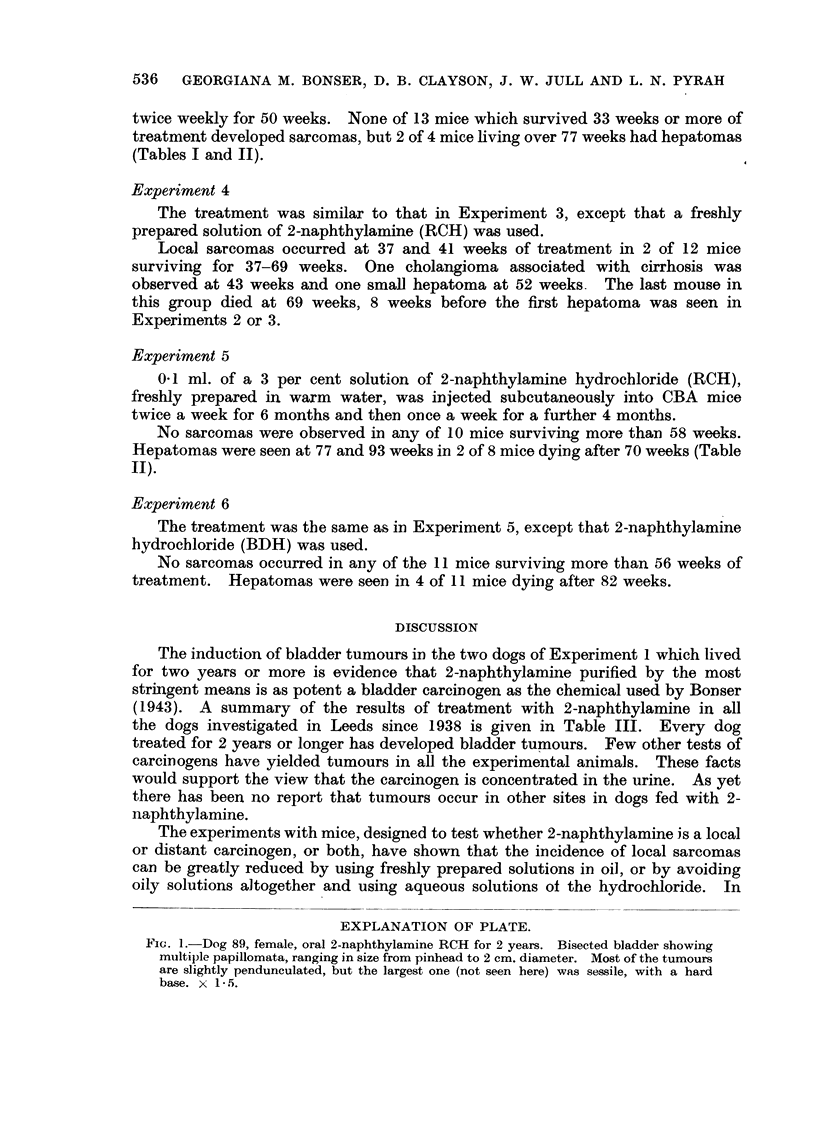

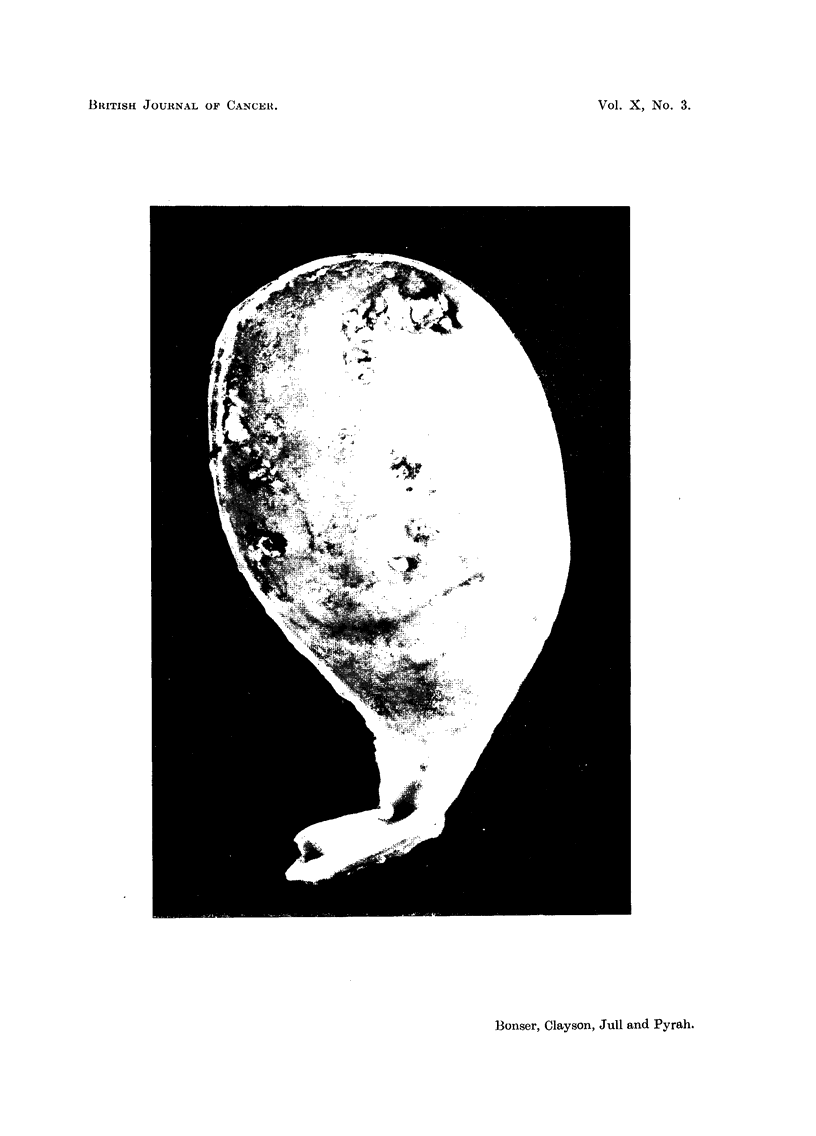

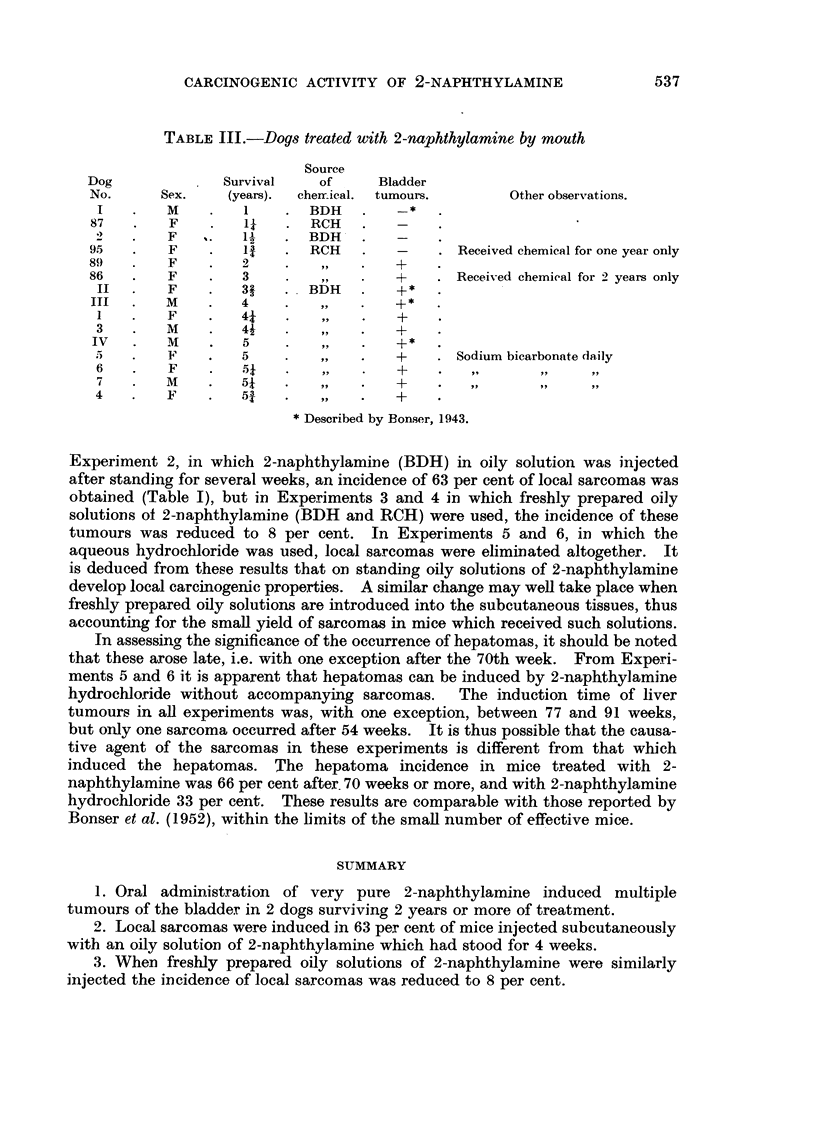

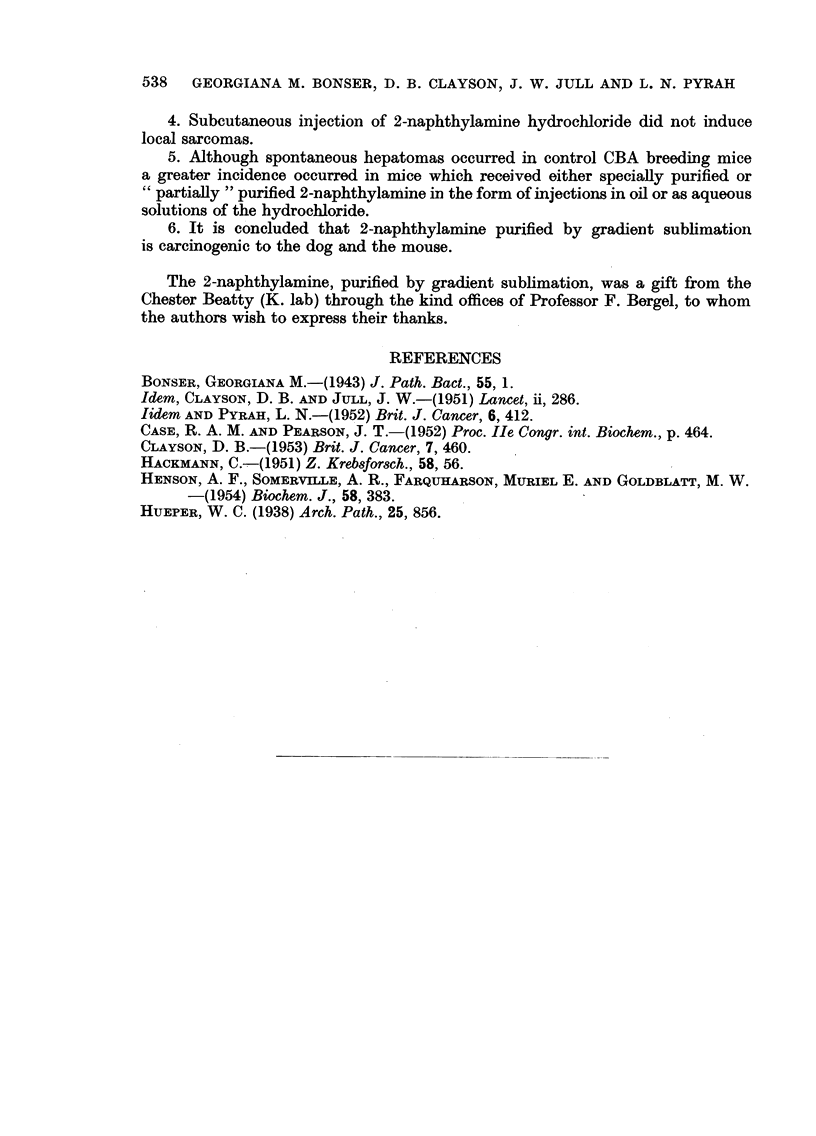


## References

[OCR_00478] BONSER G. M., CLAYSON D. B., JULL J. W., PYRAH L. N. (1952). The carcinogenic properties of 2-amino-1-naphthol hydrochloride and its parent amine 2-naphthylamine.. Br J Cancer.

[OCR_00482] CLAYSON D. B. (1953). A working hypothesis for the mode of carcinogenesis of aromatic amines.. Br J Cancer.

[OCR_00485] HENSON A. F., SOMERVILLE A. R., FARQUHARSON M. E., GOLDBLATT M. W. (1954). Metabolism of bladder carcinogens; the metabolic path of 2-[8-14C]naphthylamine in the rat.. Biochem J.

